# My nutrition index: a method for measuring optimal daily nutrient intake

**DOI:** 10.1186/s40795-022-00497-9

**Published:** 2022-02-21

**Authors:** Stefanie A. Busgang, Ashley J. Malin, Chris Gennings

**Affiliations:** 1 Department of Environmental Medicine and Public Health, Icahn School of Medicine at Mount Sinai, New York, USA; 2 Keck School of Medicine of USC, 2001 N Soto St., Los Angeles, California USA

**Keywords:** Micronutrients, Nutritional epidemiology

## Abstract

**Background:**

Adequate nutrition is essential for individual and population level health. However, determining adequacy of daily nutrient intake in research studies is often challenging given the unique nutritional needs of individuals. Herein, we examine construct, predictive, criterion, content, and concurrent validity of a dietary analytic tool – My Nutrition Index (MNI) for measuring nutrient intake in relation to personalized daily nutrient intake guidelines. MNI gauges adequacy of an individual’s daily nutrient intake based on his or her unique demographic and lifestyle characteristics. MNI accounts for potential adverse effects of inadequate and excess nutrient consumption.

**Methods:**

MNI, calculated based on 34 nutrients, provides an overall index score ranging from 0 to 100, with higher scores reflecting a more nutritious diet. We calculated MNI scores for 7154 participants ages 18-65 in the National Health and Nutrition Examination Surveys (2007-2014) by using average nutrient intakes from two 24-h dietary recalls. Survey-weighted binary logistic regression models were used to assess associations between MNI scores and obesity, depression, health perceptions, and past or present cardiovascular disease.

**Results:**

Higher MNI scores were associated with lower prevalence of self-reported cardiovascular disease (OR = 0.69, CI: 0.52, 0.92, *p* = 0.012), depression (OR = 0.76, CI: 0.65, 0.90, *p* < 0.001), and obesity (OR = 0.92, CI: 0.87, 0.99, *p* = 0.016), as well as more favorable health perceptions (OR = 1.24, CI: 1.13, 1.37, *p* < 0.001).

**Conclusions:**

MNI provides an individualized approach for measuring adequacy/sufficiency of daily nutrient intake that can validly be employed to assess relationships between nutrition and health outcomes in research studies.

**Supplementary Information:**

The online version contains supplementary material available at 10.1186/s40795-022-00497-9.

## Background

Adequate nutrition is essential for individual and population-level health [1–4], and better understanding the role of dietary nutrient quality in health outcomes can facilitate health improvements. However, capturing the nutritional value of a comprehensive diet in population-based studies is often challenging because nutritional guidelines vary among individuals based on their demographics, unique physiology, health status and physical activity level [5].

There are several different methods commonly implemented for measuring quality and/or adequacy of daily nutrient intake in research studies. These include: calculating indices such as the Healthy Eating Index (HEI) [6–9], measuring adherence to diets or nutritional recommendations empirically related to more favorable health outcomes, such as the Mediterranean Diet (MeDi), Dietary Approach to Stop Hypertension (DASH) or American Heart Association diet and lifestyle recommendations [10–12], or applying outcome-independent statistical approaches (i.e. factor analysis or cluster analysis) to characterize quality of daily nutrient intake from food frequency questionnaire or dietary recall data [13]. While each method provides valuable information on nutritional intake, these methods do not account for individual characteristics that influence daily nutrient intake guidelines, such as age, smoking status, disease states and/or physical activity level for example.

My Nutrition Index (MNI) provides an individualized and comprehensive approach to measuring adequacy and optimality of daily nutrient intake. The metric was shown to have predictive validity by demonstrating well-established associations between nutrition during pregnancy on birthweight and cognitive function in children [14]. MNI measurements are based on an individual’s unique nutritional needs (i.e., content validity), according to the Dietary Reference Intakes, developed by the National Academies of Sciences, Engineering and Medicine [15]. MNI accounts for potential adverse effects of inadequate and excess consumption of certain macro- and micro-nutrients by assigning the highest score per nutrient when the nutrient intake is within the recommended level and assigning lower scores the further from the recommended level [16–19]. Herein, we describe the characteristics of MNI and explore the distribution of MNI scores in the US adult population. First, we further examined the predictive validity of the metric by examining well-established associations between nutrition, using MNI scores, and population-level health outcomes including obesity, depression, health perceptions, and past or present cardiovascular disease among adults in the US. We expected that MNI scores would validly quantify nutritional adequacy and thus be associated with more favorable health outcomes in all measured domains. Second, we examined concurrent validity by comparing distributions of MNI scores between men and women, and between smokers and nonsmokers. Third, we examine content and construct validity by assessing the correlation between the MNI index and energy-adjusted individual nutrients. Fourth, we examined criterion validity by comparing the associations between MNI and our selected health outcomes to the associations of HEI and the same health outcomes to determine if our measure is similar to the current gold standard for population studies. Lastly, we examined convergent and construct validity by examining the correlation between MNI and HEI.

## Methods

### Participants

Participants included 18-64 year-olds participating in The National Health and Nutrition Examination Survey (NHANES) cycles 2007-2014 [20]. Adults who had two complete, valid, 24-h dietary recall, as well as complete covariate data were eligible for inclusion in analysis. Additionally, we only included participants who indicated that their 24-h dietary recall was consistent with their typical eating patterns and we excluded participants who indicated that they were on a special diet (because we could not determine their length of time on that diet). Additionally, we limited analyses to participants whose dietary recall energy values were within 3 times of their target calories based on their body size, sex, physical activity, and age (i.e., 0.33 < energy/target calories < 3.0). Ramirez-Silvia et al. [21] suggest limiting analyses to energy values within 50% of target calories, however we expanded this range to account for potential extreme increases and/or decreases in caloric intake that may correspond with the presence of depressive episodes, obesity and/or cardiovascular disease [22, 23]. There were 7154 participants who met inclusion criteria. After applying survey weights provided by NHANES, the overall sample of 7154 represented approximately 133 million adults in the United States. For analyses including depression and cardiovascular disease outcomes, we further excluded participants who were taking medication to treat that particular health condition. Specifically, we excluded participants who reported taking psychotropic medication, (i.e. anti-depressants, anti-convulsants, and antipsychotics) because people taking these medications may appear less depressed according to the depression screener, thus mis-representing their health condition The resulting sample size for testing the association between MNI scores and depression was 6105 (weighted N = ~ 73.7 million). Similarly, when examining associations between MNI scores and cardiovascular disease, we excluded participants who were taking cardiovascular medication (e.g. antiarrhythmic agents, diuretics, vasopressors, etc.), resulting in a sample size of 5860 (weighted *N* = ~ 72.6 million). We did so because participants taking these medications may be doing so for preventative reasons which may impact their cardiovascular health independently of nutritional intake. Lastly, self-reported pregnant women were excluded from the associations assessing obesity, since the increased weight gain from pregnancy would result in misclassification of these women as obese. A flow chart indicating inclusion and exclusion criteria is shown in Fig. S1.

### My nutrition index

My Nutrition Index (MNI) is a novel index that measures nutritional value of a specified daily diet based on the foods consumed in terms of how closely it adheres to the Institute of Medicine’s nutritional guidelines. It provides an overall index score ranging from 0 to 100, with higher scores reflecting a more nutritious diet. MNI is calculated based on quantification of 34 macro- and micro-nutrients that are recommended to satisfy an individual’s daily nutritional needs and accounts for caloric intake and alcohol, sugar, and caffeine consumption [15–19]. It assigns higher scores for micro- and macro-nutrient concentrations that fall within the recommended intake ranges provided by the Dietary Reference Intakes, developed by the National Academies of Sciences, Engineering and Medicine [16–19, 24–27]. Often, these guidelines are based on recommended dietary allowances (RDAs) but in the absence of an RDA they report the Acceptable Macronutrient Distribution Range, adequate intake, or tolerable upper limit where appropriate. Caffeine recommendations are based on the Food and Drug Administration upper limit of 400 mg/day for health adults [28]. It assigns lower scores if intake for a given nutrient deviates from this optimal range (i.e. deficient or excess intake). For example, a pre-adolescent between the ages of 9-14 would have a perfect score for zinc if his or her daily intake was 8 mg. The upper limit of zinc for this age group is 23 mg/d so any concentration greater than 23 would result in a lower score for zinc. Something less than 8 would also result in a lower score. For a nutrient such as potassium, the recommended level for adults is 4700 mg/d with no upper limit. Any level above 4700 would result in a perfect score and lower levels result in a lower score. The final score is calculated as the geometric mean across nutrients. Thus, a perfect MNI score would be obtained if adequate intake of all nutrients is met through diet.

MNI also incorporates dietary restrictions, individual characteristics (i.e. age, height, weight, sex, etc), activity level, and health behaviors into its calculations. Specifically, MNI can accommodate dietary recommendations based on smoking status, presence of hypertension, pregnancy, lactation, and need for a low fat, low sugar, or high protein diet, as well as any other condition for which there are special recommendations. For the current analysis, age, height, weight, sex, activity level, hypertension, pregnancy, and smoking were accounted for, while other characteristics were not because they were not available in the current dataset. In other contexts when these variables are available, the optimal ranges for specific nutrients can be adjusted if the participant has a dietary restriction or condition. Participants on special diets were excluded, as mentioned previously, since there is no information about what the special diet is or how long they have been on it. Activity level was estimated using self-reported questions related to recreational and work activity. A 5-point scale was created, with 5 being the highest activity level, based on the following responses: 5 If an individual responded to “yes” to both vigorous recreational activity and work activity (paq650 and paq605), 4 if an individual responded “yes” to only one vigorous type of activity, 3 if an individual did not respond to “yes” to either vigorous activities but did respond “yes” to both moderate recreational and work activity (paq655 and paq620), 2 if an individual did not respond “yes” to either vigorous activities but did respond “yes” to only one of the moderate types of activities, and 1 was assigned otherwise. Hypertension status, pregnancy, and smoking status were determined by self report (bpq020, RIDEXPRG, and smq020 respectfully). Therefore, MNI is calculated per participant based on published or clinical guidelines for recommended target nutrient intake ranges specific to their characteristics [15].

The following nutrients are included in MNI:

- macronutrients: protein, carbohydrates, total fat, saturated fat, monounsaturated fat, polyunsaturated fat, energy, fiber, cholesterol;
- vitamins: A, thiamin (B1), riboflavin (B2), niacin, pantothenic acid (B5), B6, folate (B9), B12, C, D, E and K;
- minerals: calcium, chloride, iron, magnesium, manganese, phosphorus, sodium, potassium, selenium, zinc; and
- other: alcohol, sugar and caffeine.

In addition to considering the degree to which nutrients fall within an individual’s recommended range, the MNI score incorporates the degree of agreement between target (i.e. recommended) caloric intake and observed diet-based calories. To determine total daily energy needs per individual, MNI utilizes the Mifflin-St Jeor equations for basal metabolic rate (BMR) which are functions of sex, weight, height and age multiplied by a 5-level physical activity factor. The lowest level is multiplied by 1.20, the second level by 1.375, the third level by 1.55, the fourth level by 1.725, and the highest level by 1.90 [29].

### Dietary nutrient intake

Dietary nutrient intake was assessed via two 24-h dietary recalls within a 3-10-day period. Dietary recalls were administered during an in-person and telephone interview respectively using the Automated Multiple Pass Method data collection tool developed by the United States Department of Agriculture [30] . Participants were asked to report the foods and beverages that they consumed over the past 24-h and to indicate whether these foods were characteristic of their regular eating patterns (i.e. “*Was the amount of food that you ate yesterday much more than usual, usual, or much less than usual*?’). Nutrient levels from food items are obtained via the Food and Nutrient Database for Dietary Studies (FNDDS) [31] The National Center for Health Statistics also determined whether 24-h dietary recalls were reliable. The average nutrient intake from both reliable and typical recalls were included in analyses. HEI scores were calculated using a SAS macro for the 2015 version of HEI provided by the National Cancer Institute (https://epi.grants.cancer.gov/hei/sas-code.html).

### Health outcomes

Though dietary patterns have been linked to many physical and mental health outcomes, for the purpose of this paper we selected four health outcomes shown to be related to nutrition in prior research: health perceptions, depression, obesity, and cardiovascular disease [32–39]. The question regarding self-perceived health asked: *“Would you say (your/Study Participant’s) health in general is*. *. .excellent, very good, good, fair or poor?”.* There were two variables reflecting self-perceived health: one that was measured via a home interview and one that was measured during the mobile examination center (MEC) visit. We utilized the variable measured from the home interview, because it had no missing data while the variable measured during the MEC visit had missing data for 762 participants.

Depressive symptoms were measured via the Patient Health Questionnaire (PHQ-9) during the MEC visit using a computer-assisted personal interview system. The PHQ-9 incorporates DSM-IV depression diagnostic criteria and has been validated for use in clinical and research settings [40–42]. It is a 9-item self-report measure designed to determine the frequency of depressive symptoms over the past 2 weeks. Each item receives a score between 0 and 3 and the maximum score attainable is 27. A score above 10 reflects the presence of self-reported depressed mood.

Obesity was defined according to the World Health Organization criteria of Body Mass Index (BMI) = > 30 kg/m^2^ [43]. Height and weight were measured during the MEC visit and BMI was calculated as weight in kilograms divided by height in meters squared, rounded to one decimal place. Though several studies have shown that BMI may not be an optimal measure of adiposity, Nutall et al. (2015) reviewed several studies which showed that being a little slightly overweight, according to BMI ranges, results in lower morbidity and mortality. Nutall also points to several studies where BMIs greater than 30 were an indication of higher mortality [44].

Lastly, to assess the presence of current or past cardiovascular disease, participants over the age of 20 years old were asked 5 separate questions regarding whether they had ever been diagnosed with the following conditions: *congestive heart failure, coronary heart disease, angina pectoris, heart attack,* or *stroke*. Participants who indicated that they had or have any of these conditions were considered to have past and/or current cardiovascular disease respectively [45].

### Covariates

Covariates were selected a priori based on being empirically related to nutrient intake/metabolism and health outcomes of interest in prior studies [46–53]. They included: age, sex, race/ethnicity, education level and healthcare use. Education level was defined by a 5-level categorical variable ranging from less than 9th grade to college graduate or above. We adjusted for healthcare use as this may be a proxy for other health behaviors that may influence health outcomes and it may also be associated with dietary nutrient intake.

### Statistical analysis

We applied dietary sampling weights, specifically the two-day weight, using survey procedures in SAS 9.4 to account for survey non-response, the clustered sample design, over-sampling, post-stratification, sampling error, differential allocation by day of the week for the dietary intake data collection, and to permit generalization to non-institutionalized adults in the United States population [54].

We calculated binary variables for each health outcome to examine its relationship with MNI scores dichotomously. For health perceptions, we computed a variable of favorable perceived health = 1 and unfavorable perceived health = 0, to compare those who rated their health as excellent, very good, and good (1) to those who rated their health as fair or poor (0) [55]. For remaining outcomes, we computed a binary variable of depressed mood (i.e., PQH-9 score = > 10) = 1 and non-depressed mood ((i.e., PQH-9 score < 10) = 0; obese (BMI = > 30) = 1 and non-obese (BMI < 30) = 0; and current or past cardiovascular disease: yes = 1, no = 0. Four survey-weighted multivariable logistic regression models examined associations between MNI scores and each binary health outcome after adjustment for covariates. We converted MNI scores to a 10-point scale for regression analyses to better capture meaningful changes in dietary quality. Whereas nutrient intake may change slightly from day to day (i.e. captured by a few points change in MNI score on a 100-point scale), a 10-point change is more likely to reflect meaningful differences in nutritional intake. Spearman correlations were used to assess the correlation between MNI and individual nutrients, adjusted for energy. We also conducted analyses comparing distributions of MNI scores to HEI scores, as HEI is a valid and reliable nutrient measurement index that is widely used in research studies [56]. Different from MNI, HEI is based on food consumption patterns recommended by the US Dietary Guidelines (2015-2020). Spearman correlations were used to assess the correlation between MNI and HEI. Lastly, we examined associations between MNI and HEI scores, and compared their associations with the health outcomes examined herein. HEI scores range from 0 to 100 but, as with MNI scores, were converted to a 10-point scale for ease of interpretation. Additionally, for comparisons of associations of MNI and HEI with health outcomes, we centered and scaled MNI and HEI scores to account for differences in their distributions.

## Results

**Fig. 1:**
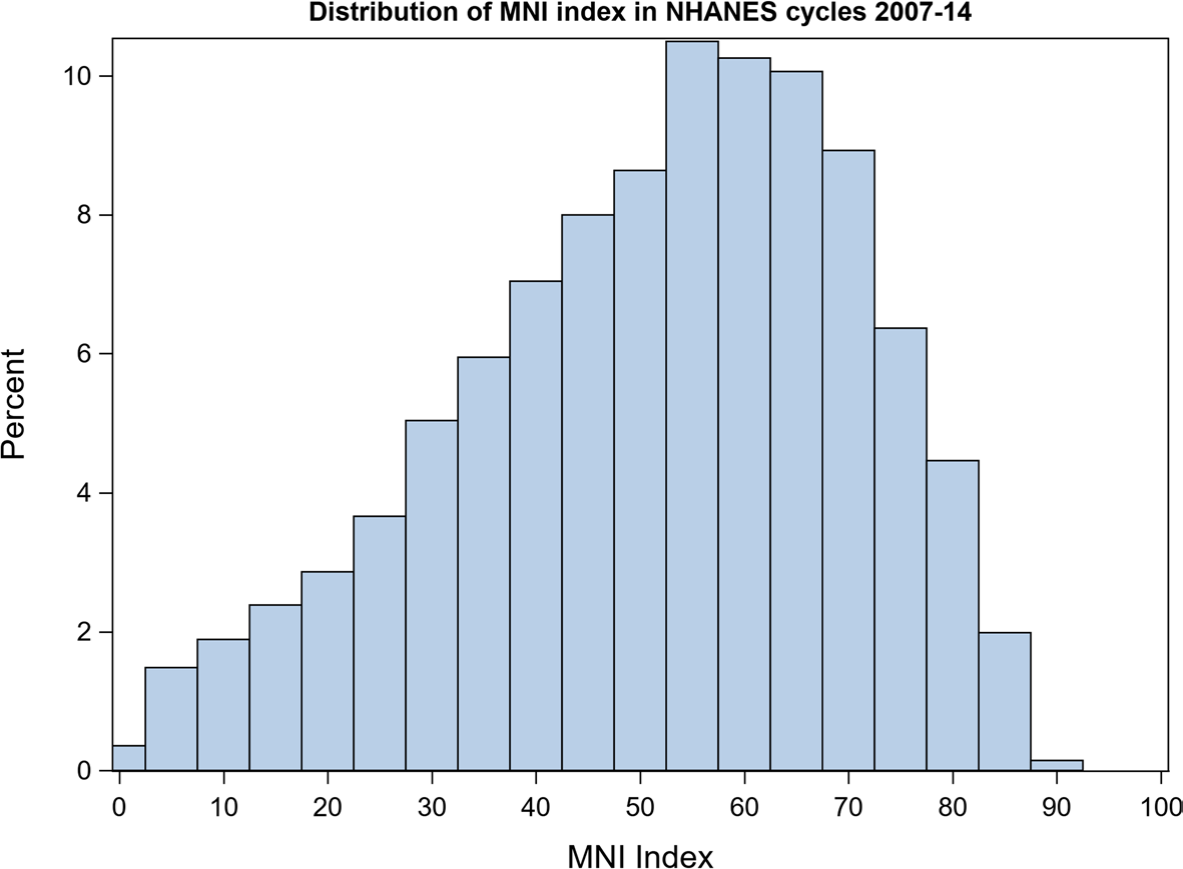
MNI distributions among participants aged 18-65 from NHANES cycle 2007-2014

**Fig. 2:**
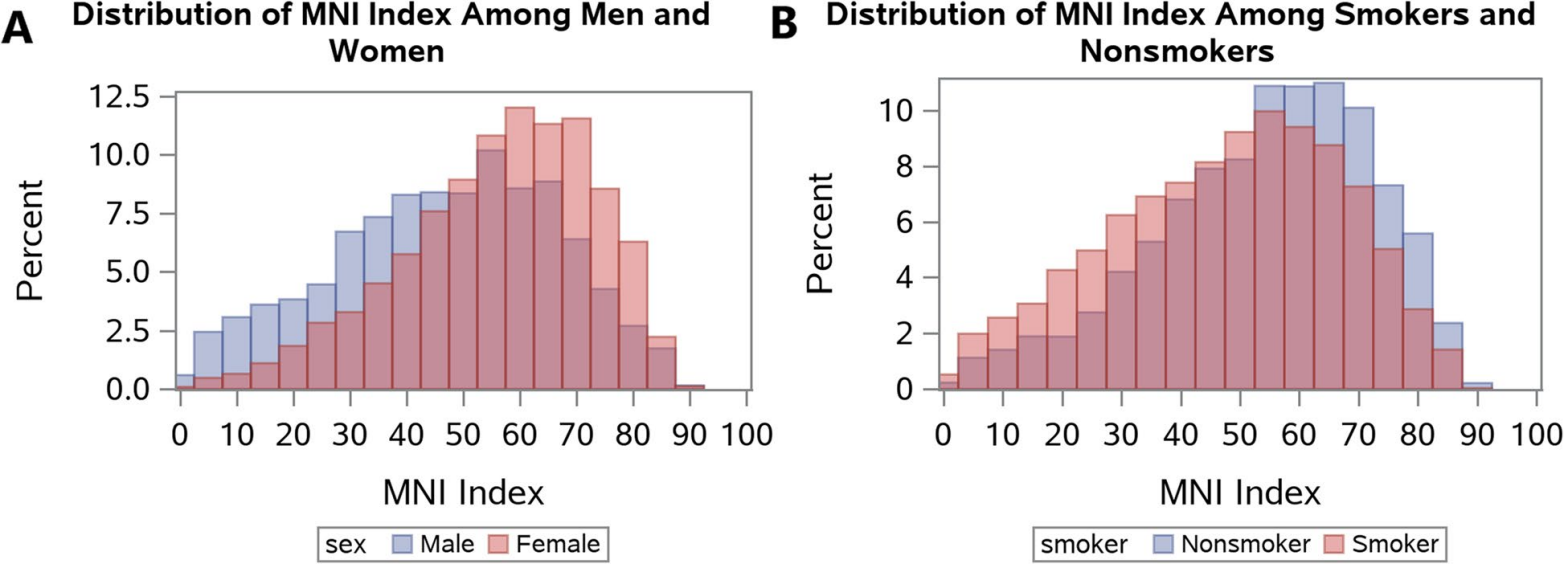
A comparison of MNI distributions among (**a**) men and women and (**b**) smokers and nonsmokers from NHANES 2007-2014

**Fig. 3:**
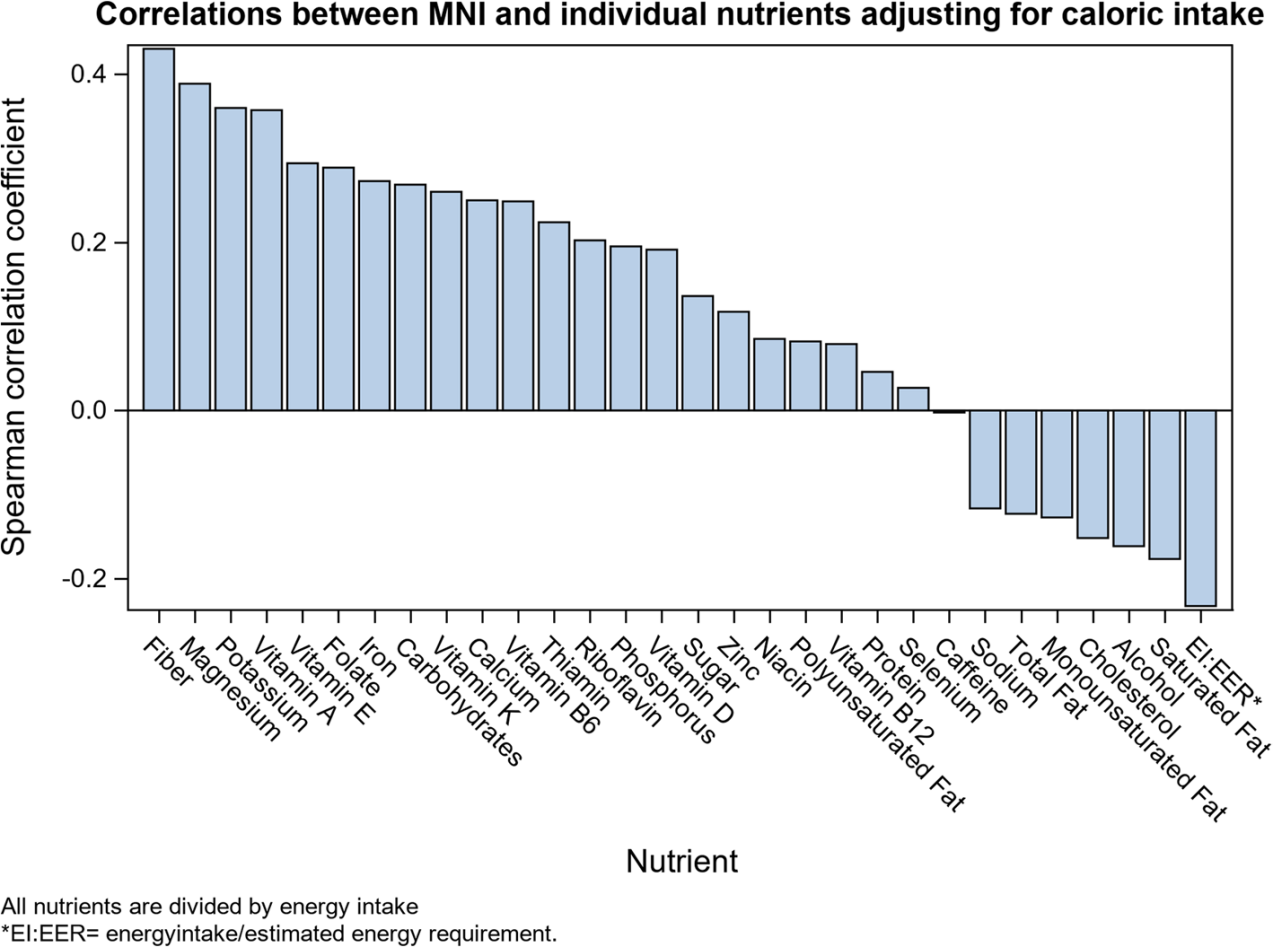
Pearson correlations of the MNI index with each individual nutrient, adjusted for caloric intake

Descriptive statistics for socio-demographic characteristics and health outcomes are presented in Table 1. The prevalence of each health outcome remained consistent across all NHANES cycles examined. MNI scores were approximately normally distributed, with the majority of participants having MNI scores between 50 and 70 and few participants having very low (i.e. < 20) or very high (i.e. > 80) scores (Fig. 1). Distributions of MNI scores stratified by sex and smoking status are depicted in Fig. 2. Mean MNI scores were, on average, approximately 10 points higher for women than for men and for non-smokers than smokers. Spearman correlations between MNI scores and individual nutrients, adjusted for caloric intake resulted in positive correlations for all vitamins and most nutrients that promote health (i.e. “good nutrients”) and negative correlations for most nutrients that tend to detract from health when consumed in excess (Fig. 3).

**Table 1:** Sociodemographic characteristics of the study sample from NHANES cycles 2007-2014

	Unweighted Frequency (*n* = 7154)	Unweighted %	Weighted % (*N* = 87,869,105)
**Sex**			
Male	3622	50.6	50.9
Female	3532	49.4	49.1
**Education**			
Less than 9th grade	564	7.9	4.0
9-11th grade	957	13.4	9.9
High school grad/GED or equivalent	1579	22.1	21.4
Some college or Associates of Arts	2101	29.4	31.3
College graduate or above	1953	27.3	33.4
**Race**			
Mexican American	1154	16.1	8.7
Other Hispanic	684	9.6	4.9
Non-Hispanic White	3327	46.5	71.0
Non-Hispanic Black	1233	17.2	8.8
Other race/Multi-racial	756	10.6	6.7
**Pregnant (females only)**			
No	3443	97.5	97.4
Yes	89	2.5	2.6
**Smoker**			
No	5539	77.4	79.1
Yes	1614	22.6	20.9
Missing	1		
**Routine use of healthcare**			
No	1402	19.6	17.5
Yes	5752	80.4	82.5
**Self-reported Health Status**			
Good, Fair, Poor	1283	17.9	13.3
Excellent, Very good	5866	82.1	86.7
Missing	5		
**Self-reported Depression**			
No	6188	92.8	93.4
Yes	481	7.2	6.6
Missing	485		
**Obesity (elevated BMI)**			
< 30	4636	65.6	67.2
≥ 30	2429	34.4	32.8
Missing	89		
**Cardiovascular disease**			
No	6865	96.1	96.7
Yes	281	3.9	3.3
Missing	8		
	**Unweighted Mean**	**Unweighted SE**	**Weighted Mean (SE)**
Age	42.2	13.0	41.8 (0.3)
Ratio of family income to poverty (PIR)	2.62	1.70	3.10 (0.1)
My Nutrition Index	50.8	18.6	51.3 (0.4)
Healthy Eating Index	53.5	16.9	53.5 (0.3)

Associations between MNI scores and health outcomes, after adjustment for covariates, are presented in Table 2. Higher MNI scores were associated with significantly lower odds of experiencing past or present CVD (OR = 0.69, 95% CI: 0.52-0.92, *p* = 0.012), reporting symptoms of depression (OR = 0.76, 95% CI: 0.65-0.90, *p* < 0.001), or being obese (OR = 0.92, 95% CI: 0.87-0.99, *p* = 0.016). Higher MNI scores were associated with significantly higher odds of reporting more favorable health perceptions (OR = 1.24, 95% CI: 1.13-1.37, *p* < 0.001).

**Table 2:** Associations of a 10-point change in MNI score with health outcomes

	OR	95% CI	*p*-value
Obesity (*n* = 7112)	0.92	0.87, 0.99	0.016*
Self-reported health (*n* = 7149)	1.24	1.13, 1.37	< 0.001***
Depression (*n* = 6105)	0.76	0.65, 0.90	< 0.001***
CVD (*n* = 5860)	0.69	0.52, 0.92	0.012*

*CVD* Cardiovascular diseases; Participants were adults aged 18-65 in NHANES 2007-2014; analyses were adjusted for age, sex, race, education, and healthcare access; sampling weights were applied for regression analyses; Weighted Ns: obesity, *N* = 87,314,021; self-reported health, *N* = 87,836,410; depression, *N* = 73,671,431; CVD, *N* = 72,605,406

*p < 0.05; **p < 0.01; ***p < 0.001

**Fig. 4:**
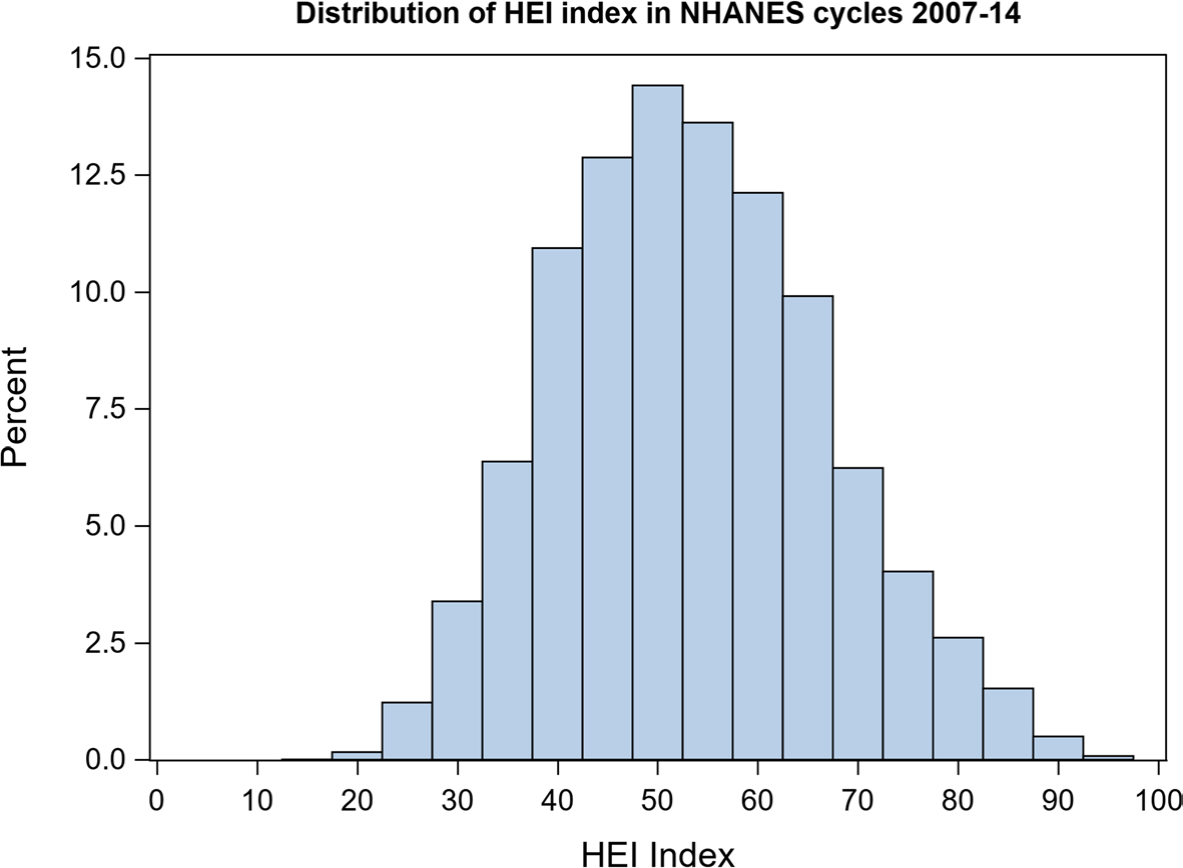
HEI distributions among participants aged 18-64 from NHANES cycle 2007-2014

**Fig. 5:**
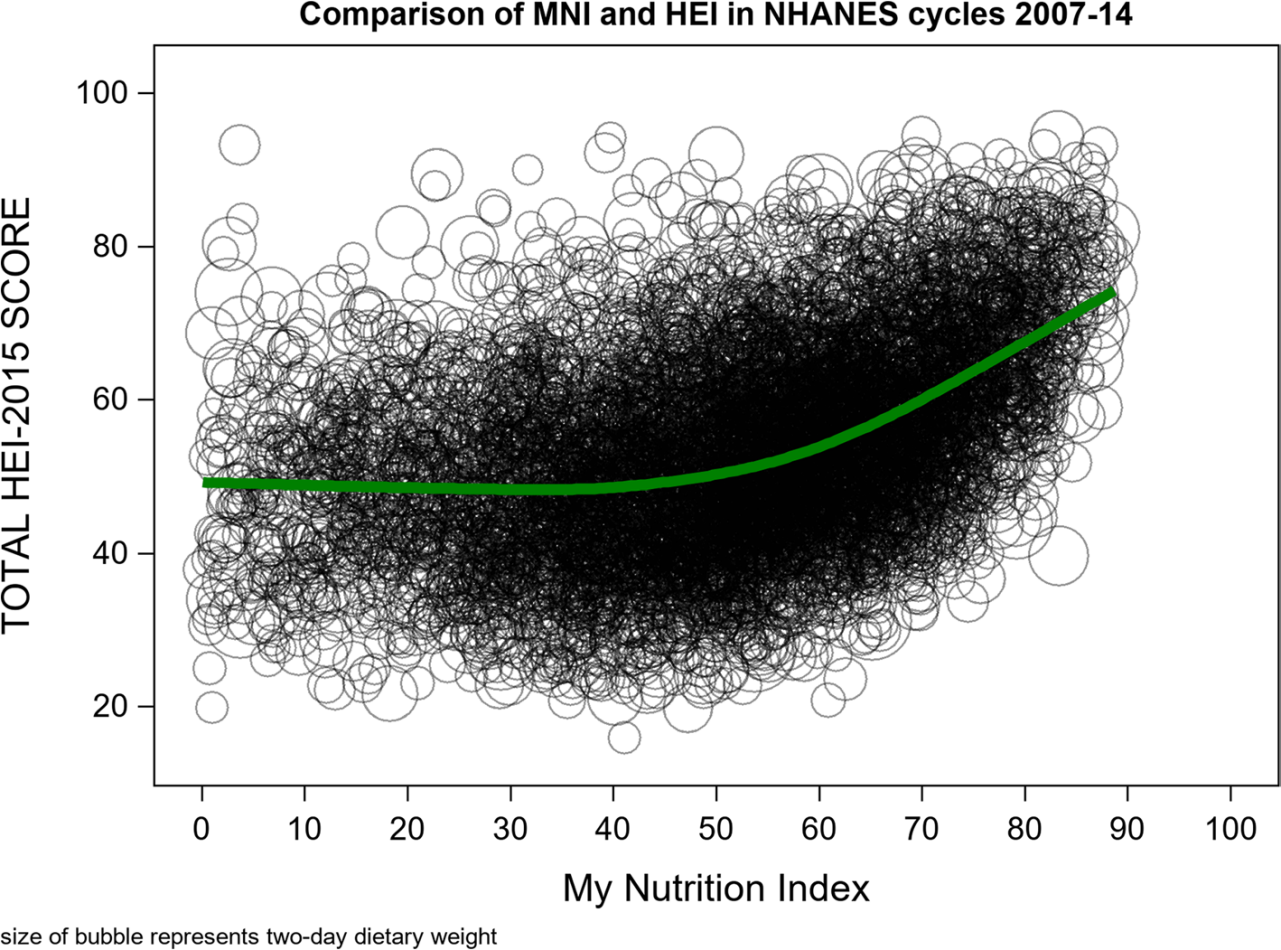
Comparison of My Nutrition Index and the Healthy Eating Index from NHANES cycle 2007-2014

Similar to the distribution of MNI scores, the distribution of HEI scores was approximately normal; however, it had smaller variance **(**Fig. 4**)**. MNI and HEI scores were moderately positively correlated (*r* = 0.39, *p* < 0.001; Fig. 5); however, a curvilinear association was evident graphically such that the association is closer to identity for higher scores (i.e. above 50). Survey-weighted logistic regression of associations between HEI scores and health outcomes, after covariate adjustment, yielded similar associations to those found between MNI scores and health outcomes (Table 3). However, the magnitude of association between HEI scores and obesity was larger than between MNI scores and obesity.

**Table 3:** Associations between HEI scores and health outcomes

	OR	95%CI	*p*-values
Obesity (*n* = 7112)	0.74	0.69, 0.80	< 0.001***
Self-reported health (*n* = 7149)	1.23	1.11, 1.36	< 0.001***
Depression (*n* = 6105)	0.71	0.61, 0.82	< 0.001***
CVD (*n* = 5860)	0.52	0.37, 0.74	< 0.001***

*CVD* Cardiovascular diseases; Participants were adults aged 18-65 in NHANES 2007-2014; analyses were adjusted for age, sex, race, education, and healthcare access; sampling weights were applied for regression analyses; Weighted Ns: obesity, *N* = 87,314,021; self-reported health, *N* = 87,836,410; depression, *N* = 73,671,431; CVD, *N* = 72,605,406

*p < 0.05; **p < 0.01; ***p < 0.001

## Discussion

It is well established that adequate dietary nutrition is essential for health [57]. However, relationships between dietary nutrient intake and specific health outcomes are often complex and difficult to measure [5, 58]. My Nutrition Index (MNI) quantifies dietary nutritional value based on an individual’s characteristics, according to established dietary guidelines. It provides an overall index score that is adjusted for body size, age, sex, physical activity level, health status, and certain behaviors (see example in Appendix).

We examined MNI scores among adults participating in NHANES from 2007 to 2014 who reported two 24-h dietary recalls that were typical of their daily diet. We found that MNI scores were normally distributed with a mean of 50.8 and a median of 52.8. This suggests that most US adults tend to eat moderately nutritious diets. Consistently, findings from a study on dietary patterns among adults participating in NHANES from 2011 to 2012 found that just over 50% followed intermediately healthy diets [59]. Interestingly, we also found that MNI scores were higher among women and non-smokers, compared to men and smokers respectively. These findings are also consistent with prior studies showing that women and non-smokers tend to report adherence to more healthful dietary practices [60, 61]. For example, women have been shown to be more compliant with the Mediterranean diet [39], and non-smokers tend to consume more fruits and vegetables [62–64].

We also found that higher MNI scores detected anticipated and important associations between healthy eating and more favorable health outcomes. Specifically, a 10-point increase in MNI score was associated with 0.83 (17%) lower odds of having past or present CVD, 0.87 (13%) lower odds of reporting feeling depressed, 0.97 (3%) lower odds of being obese, and 1.08 (8%) higher odds of reporting excellent or very good health. These findings are consistent with prior studies showing that obese adults are more likely to suffer from micronutrient inadequacies, and that consuming a more nutritious diet may decrease the risk of depression, CVD and death from CVD [36, 65–70]. Thus, MNI captures associations between dietary nutritional adequacy and health outcomes in population-based studies in the expected directions.

We also compared distributions of MNI and HEI scores, as well as their relationships with health outcomes, as the HEI is a widely used, valid and reliable measure of nutritional intake in population-based studies. HEI was developed by the USDA to measure alignment between specific food group choices and USDA dietary guidelines [56]. Thus, unlike MNI, it does not quantify micronutrients, adjust for individual characteristics, or account for potential adverse effects from excess micronutrient intake. Similar to MNI scores, we found that HEI scores among NHANES participants were normally distributed; however, MNI scores were more variable. This suggests that MNI may capture a broader spectrum of eating behaviors. Still, MNI and HEI scores were similarly associated with the health outcomes examined herein which provides additional support for the construct and criterion validity of MNI.

Despite its similarities with HEI in terms of distribution and associations with health outcomes, MNI is an innovative research tool. It is unique in that its calculations can account for any personal characteristic, lifestyle factor or health condition for which there are specific Dietary Reference Intake guidelines. For example, the Dietary Reference Intakes recommend that smokers should have higher vitamin C intake [71], given that smoking can affect vitamin C metabolism [72–75]. Therefore, if two people with otherwise equal characteristics eat the exact same foods in a given day and have average Vitamin C intakes, yet one is a smoker, the non-smoker would have a higher MNI score. To our knowledge, HEI does not take in to account these additional lifestyle behaviors. Additionally, lactating women have higher selenium and potassium guidelines [76] and this is reflected in their MNI scores. Pick et al. examined HEI in pregnant women and concluded that it failed to discern micronutrient deficiencies unique to pregnancy and that a new HEI, specific for nutrient needs during pregnant should be developed [77]. Not only can the MNI index be adjusted by population level recommendations, but it can also be personalized such that an individual can work with their health care provider or nutritionist to accommodate whatever health or lifestyle deviations may be needed to determine the optimal level for that person. For example, if results of a blood test indicate that a patient has low iron, the health care provider could adjust the index so that the highest MNI score is attained only at a higher level of dietary iron, compared to someone with normal iron levels. Furthermore, in accordance with established nutritional guidelines, MNI uniquely considers excess consumption of certain nutrients aversive. For example, excess consumption of iron can increase oxidative stress and contribute to neurotoxicity [78–80]. Therefore, excess consumption and under consumption of this nutrient would lower an individual’s MNI score.

Another benefit of MNI over HEI is the ability to incorporate supplemental nutrients in addition to dietary nutrients. MNI has the capability to account for different recommendations of a nutrient, depending on its source – food or supplements. Supplemental nutrients are also important for a healthy diet. For example, iodine is an important micronutrient, especially during pregnancy. Iodine in dietary sources can be scarce so fortification and supplementation are often used. In the current study, we chose to focus on the nutrient data from dietary, beverage, and water sources only. For this reason, iodine was not included in our index due to the lack of data on dietary iodine. However, NHANES provides data on iodine from supplements and thus can be incorporated in future iterations of the index.

## Limitations

Nutritional adequacy measurements with MNI are subject to several limitations. As with other approaches, MNI measurements that rely on self-reported food intake measures, such as food frequency questionnaires or dietary recalls for example, may be subject to recall biases and/or social desirability. The nutrient values used in this analysis were based on a short-term dietary assessment metric and thus may not be reflective of long-term eating behaviors. Additionally, like HEI scores, MNI scores assess *food-based* nutritional adequacy and thus do not account for supplement intake. Therefore, they may not capture true total daily nutrient intake. Nutrient intake misclassification may bias associations with health outcomes to the null; however, in our analyses we still found significant associations. Third, while MNI accounts for a breadth of nutrients and personal characteristics, it does not account for nutrients that do not have established values for Recommended Dietary Allowance (RDA) or values of Adequate Intake when insufficient evidence is available to establish an RDA. Over time, and with more research, additional nutrients with established guidelines can be incorporated into the MNI calculations. (e.g., copper and iodine). Lastly, in examining associations between MNI and health outcomes, we utilized cross-sectional data which is limited for determining directionality of associations found. In future studies, we plan to prospectively examine whether the individualized approach to nutritional adequacy assessment of MNI can predict relationships with health outcomes over time.

## Conclusion

MNI is a novel research tool that measures nutritional value of an individual’s daily diet according to his or her personal characteristics, lifestyle factors and health status. MNI provides a dietary nutrition assessment that validly captures associations between overall dietary nutritional value and health outcomes in population-based studies.

## Supplementary Information

**Additional file 1.**

## Data Availability

The datasets generated and/or analyzed during the current study are available on the Center for Disease Control and Preventions website at https://www.cdc.gov/nchs/nhanes/index.htm.

## Abbreviations

MNI: My Nutrition Index
NHANES: National Health and Nutrition Examination Survey
HEI: Healthy Eating Index
BMI: Body mass index
RDA: Recommended Dietary Allowance
