# Supplementary material for: My nutrition index: a method for measuring optimal daily nutrient intake
Source: BMC Nutr. 2022 Feb 21;8:16. doi: 10.1186/s40795-022-00497-9 (PMC8862522; doi:10.1186/s40795-022-00497-9)
Supplement: Supplementary file 1 — Additional file 1. [file 40795_2022_497_MOESM1_ESM.docx]

**Figure S1**. Participant selection flowchart

**Participants in NHANES 2007-2014**

N=40,617

**Participants ages 18-65 years old**

N=19,097

**Two reliable and typical 24-hour dietary recalls**

N=8,384

**Data on at least on one health outcome**

N=15,136

**Complete covariate data**

N=14,170

**Not on any kind of special diet**

N=7,268

**Caloric intake is no more than 3x higher or lower than estimated target calories using Mifflin St Jeor equation**

N=7,154

**Depression:**

**Participants not taking mood stabilizers**

N=6,105

**Obesity:**

**Participants who are not currently pregnant**

N=7,112

**CVD:**

**Participants who are not taking cardiovascular medication**

N=5,860

**Self-reported health:**

N=7,149

**Appendix S1**. Example of individualized dietary needs.

The Mediterranean diet is a heart healthy eating plan, rich in vegetables (e.g., spinach, tomatoes, squash) and legumes (e.g. white beans; lentil soup), fresh fruit, olive oil, and moderate amounts of grains (e.g., wheat bread), fish and poultry [1]. Consider a hypothetical moderately active average sized young man with high daily legume and pasta intake. He may have a moderate MNI value (e.g. mid 70s) because folate, sodium and fat levels are elevated. However, by reducing sizes of his bean, lentil soup and pasta servings, for example, folate, sodium, and fat levels would decrease and his MNI score could increase to nearly 100. However, for a middle-aged, lightly active, petite woman the optimal Mediterranean diet, as described for the young man, likely has too many calories, too much sodium, folate, and total fat. Eating the same foods and portion sizes as the man, this woman’s MNI score would be only about 50, but by eliminating white beans and lentil soup entirely, for example, calories, sodium and folate would reduce; however, the consequence is also a decrease in fiber and magnesium. To compensate, an increase in spinach, tomatoes, squash and wheat bread could increase the magnesium levels, and fiber. Now consider a similarly middle-aged, petite woman who is sedentary and has a chronic health condition requiring a high protein, low fat diet with caffeine restrictions. The resulting alteration in the Mediterranean diet has too many calories and carbohydrates, too much sodium, and total fat and not enough vitamin D; the MNI only about 30. The exercise demonstrates the complexity of ‘eating right’, particularly with health-based dietary restrictions. Nutritional requirements change with individual characteristics and finding the best food choices and portion sizes that improve daily nutrition is a multifaceted process [2].

**REFERENCES**

1. Trichopoulou A, Costacou T, Bamia C, Trichopoulos D. Adherence to a Mediterranean diet and survival in a Greek population. New England Journal of Medicine. 2003;348(26):2599-608.

2. C G, A W, N H, C L, CG B. Contrasting prenatal nutrition and environmental mixtures in association with birth weight and cognitive function in children at 7 years. BMJ Nutrition, Prevention & Health. 2020.
